# Mouse Norovirus Uses Host Metabolites to Enhance Receptor Binding and Evade Immune Recognition

**DOI:** 10.18103/mra.v10i11.3270

**Published:** 2022-11-28

**Authors:** Michael B. Sherman, Alexis N. Williams, Hong Q. Smith, Christiane E. Wobus, Thomas J. Smith

**Affiliations:** 1 University of Texas Medical Branch at Galveston, Department of Biochemistry and Molecular Biology, 301 University Boulevard, Route 0645, Galveston, TX, 77555; 2 Department of Microbiology and Immunology, University of Michigan Medical School, 1150 West Medical Center Dr., Ann Arbor, MI, 48109

## Abstract

Noroviruses are the major cause of epidemic gastroenteritis in humans, causing ~20 million cases annually, resulting in more than 70,000 hospitalizations and 570–800 deaths in the United States alone. The T=3 icosahedral calicivirus capsid is composed of viral protein 1 (VP1) with three major domains: the N-terminus (N), shell (S), and C-terminal protruding (P) domains. The S domain forms a shell around the viral RNA genome, while the P domains dimerize to form protrusions on the capsid surface. The P domain is subdivided into P1 and P2 subdomains, with the latter containing the binding sites for cellular receptors and neutralizing antibodies. Mouse norovirus (MNV) is a widely used system for study of norovirus biology since we have a cell culture system, reverse genetic tools, and small animal model to eventually correlate structural information to whole animal pathology

Mouse norovirus is a surprisingly dynamic virus that switches between receptor and antibody binding structures depending upon the in-vivo environment. In the circulation, the P domain floats above the shell by more than 15Å and the P domain loops (A’B’/E’F’) at the very tip are splayed apart in an ‘open’ conformation that antibodies learn to recognize. Upon ingestion, the low pH environment with high metal and bile salt concentrations in the alimentary canal each independently trigger the P domains to rotate 90° and contract by 15 Å onto the capsid surface. This hides any epitopes at base of the P domain. During this reversible collapse, the two P domains within the dimer rotate about each other and the A’B’/E’F’ loops adopt the ‘closed’ conformation. This opens the receptor binding site while burying the epitopes at the tip of the P domain. Therefore, rather than only depending on escape mutations to block antibody binding, MNV aggressively uses host conditions to remodel itself to enhance receptor binding while blocking antibody recognition.

This review will describe the structural processes and biological consequences of the virus responding to activating host cues in the gut while these same triggers bury the epitopes presented in the circulation. This is an aggressive and unique mode of immune escape that has been subsequently shown in other viruses such as COVID-19. Therefore, a deeper understanding of the dynamic processes of virus capsids will improve vaccine design by understanding how to present the epitope conformations at the site of infection rather than what is presented to the immune system.

## Introduction

There are 11 genera in the *Caliciviridae* family of which 7 infect animals including noroviruses. Based on genomic sequences, noroviruses are further divided into 10 genogroups (GI-GX) that are further subdivided into 49 genotypes: 9 GI, 27 GII, 3 GIII, 2 GIV, 2 GV, 2 GVI and 1 genotype each for GVII, GVIII, GIX (formerly GII.15) and GX^1^. Controlling the spread of norovirus is challenging since as few as ten virions are sufficient to infect an adult^2^. Efforts to make effective norovirus vaccines have been thwarted among others by our lack of efficient and easy to use human norovirus cell culture systems limiting our understanding of the structural mechanisms of viral escape from antibody neutralization. In addition, noroviruses are constantly evolving and frequently generating new strains^3–5^ that result in worldwide epidemics^5,6^. Developing efficacious vaccines requires a structural understanding of how the virus evades the immune system. Murine norovirus (MNV1, genotype GV.1) is a powerful surrogate for the human noroviruses since it can be grown to high titers in cell culture, there is a reverse genetic system, and mice serve as a convenient animal model system.

M-cells (microfold cells) serve as the uptake site of the mucosal immune system to transport antigens from the gut lumen to underlying immune cells localized at Peyer’s patches in the lamina propria^7–9^. MNV1 hijacks M-cells for internalization and then infects cells from the adaptive and innate arms of the immune system (MΦ, dendritic, B, and T cells. A secondary site of MNV infection is Tuft cells^10^. Tuft cells function as sentinel cells in the gut, detecting pathogens and signaling the mucosal immune system. The exact role of Tuft cells in MNV infection is unclear, but it may be involved in the establishment of persistent infections. *In-vivo* tropism and infection type varies among the various MNV strains^11^.

The immunoregulatory protein CD300lf was identified as the receptor for MNV1 using genome-wide CRISPR/Cas9 in murine macrophage-like cells^12,13^. The dependence of MNV on CD300lf for attachment and infection was verified with a series of experiments showing depletion or blockade of CD300lf confers resistance to MNV^14,15^. CD300lf is a type I membrane-bound glycoprotein that modulates leukocyte function expressed on adaptative and innate immune cells and tuft cells^11,16,17^. CD300lf is functionally conserved in humans but does not serve as the proteinaceous receptor for any tested human norovirus^18^.

**Figure 1:**
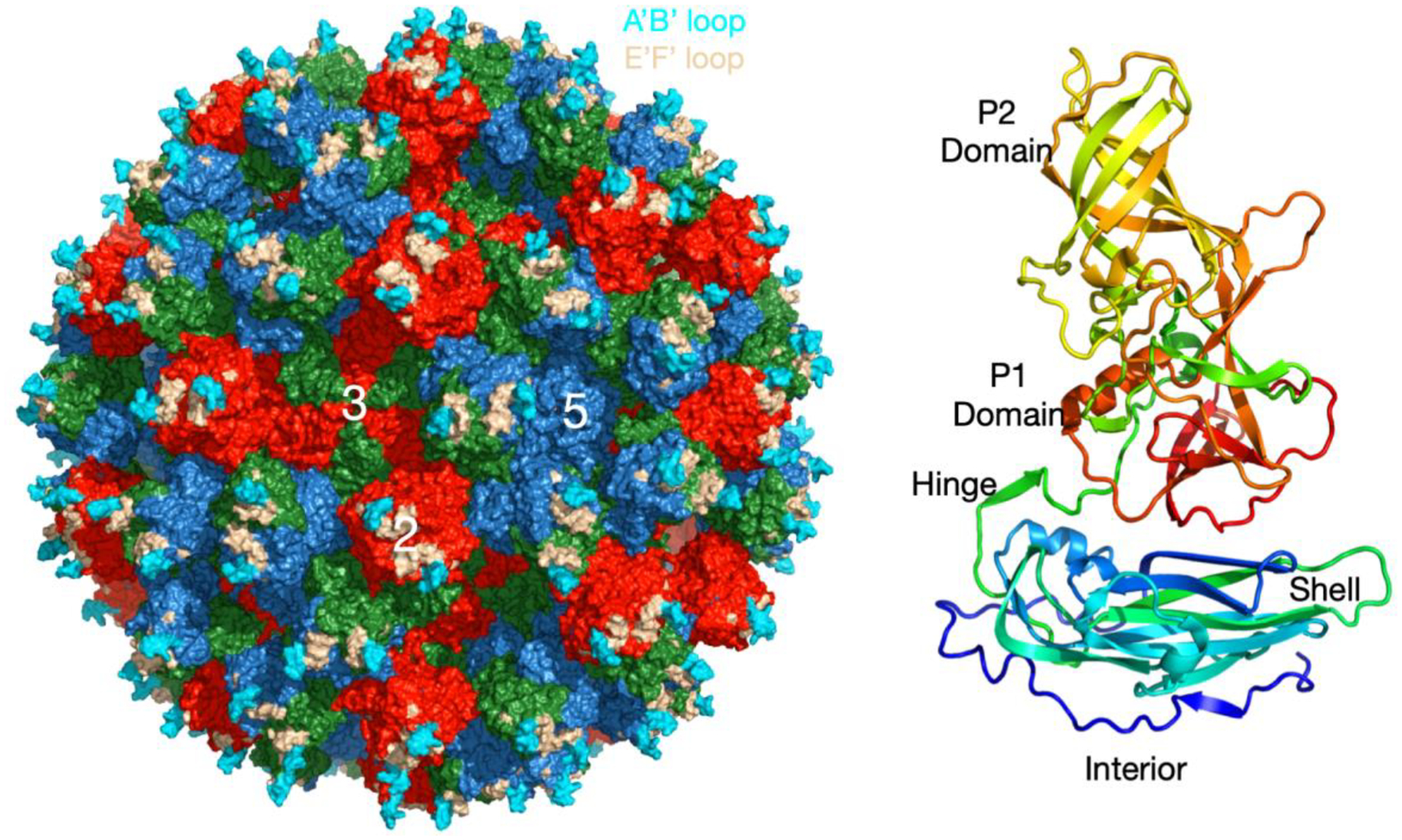
Structure of MNV. The left figure shows the surface rendering of the entire capsid with the A’B’ and E’F’ loops highlighted in turquoise and tan, respectively. The right figure is a ribbon diagram of a single copy of the capsid protein with the various domains noted.

The calicivirus capsid is composed of 180 copies of VP1 with a molecular weight of ~58 kDa. Caliciviruses are T=3 icosahedral particles (Figure 1) with 180 copies of the major capsid protein (VP1; ~58 kDa), that is divided into the N-terminus (N), the shell (S) and C-terminal protruding (P) domains^19–23^. The S domain forms a shell around the viral RNA genome and the P domains form protrusions comprised of A/B and C/C dimers. The P domain is further subdivided into P1 and P2 subdomains with the latter containing the binding sites for cellular receptors^24,25^ and neutralizing antibodies^26–28^.

## The highly flexible MNV P domain

**Figure 2:**
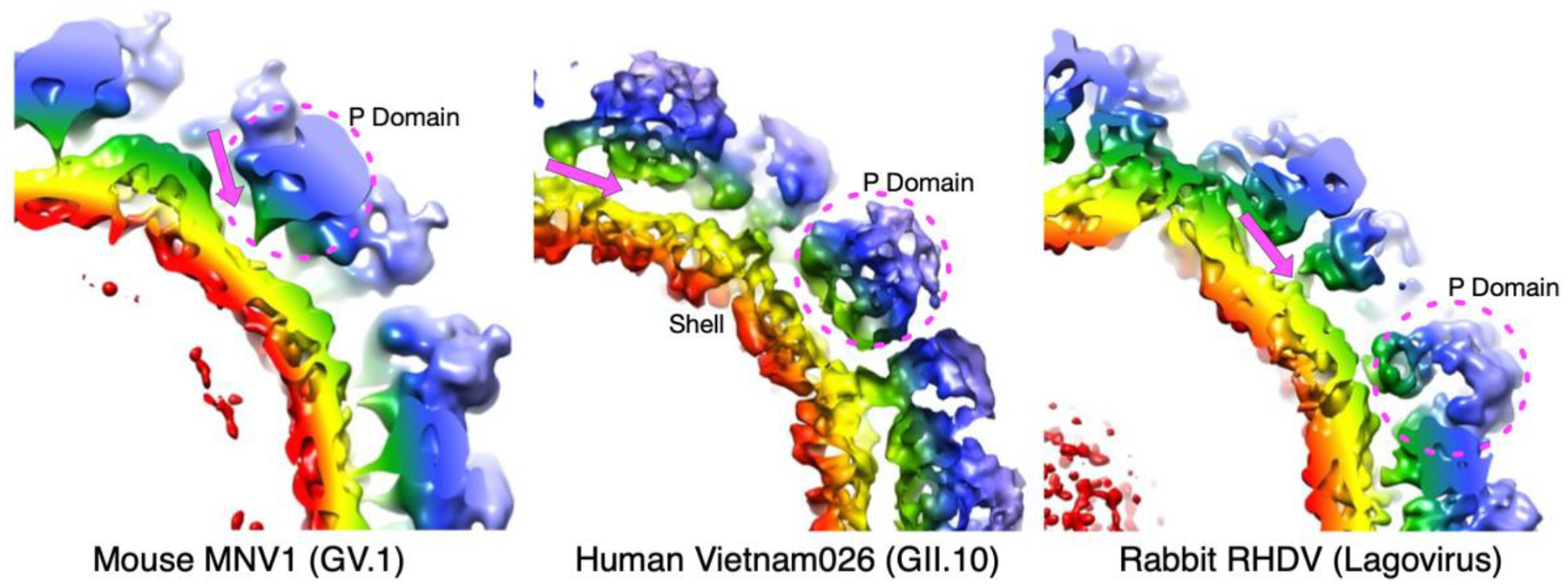
Cryo-EM structures of three different caliciviruses. All three have highly mobile P domains that are lifted well off the shell surface (mauve arrow).

Our structural studies on MNV began with a cryo-EM structure that showed the P domain to be highly mobile and ‘floating’ ~15Å above the surface of the shell^22,29–32^. This ‘floating P domain’ structure was met with skepticism since it had not been observed in any other viral capsid (Figure 2). However, we subsequently observed that the P domains of other Caliciviruses, RHDV and NV GII, were similarly highly mobile. Therefore, the fact that this ‘floating P domain’ is conserved across Calicivirus genotypes and genera^29,33,34^ gives importance to the finding and suggests a yet unknown biological function.

**Figure 3:**
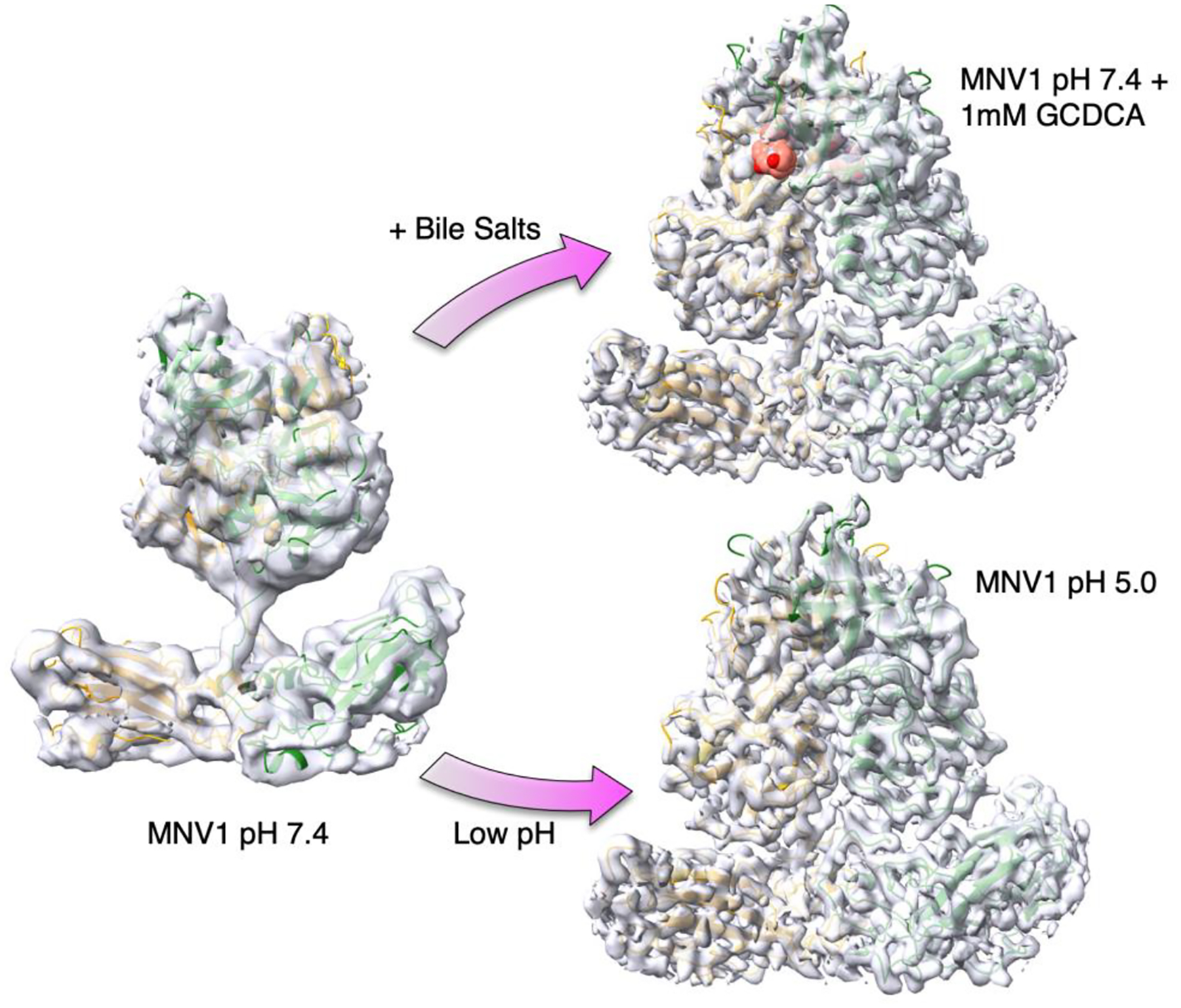
Cryo-EM image reconstructions of MNV before and after activation by low pH or bile salts.

The Virgin and Fremont labs found that the MNV receptor is CD300lf and that some bile salts (e.g. GCDCA) enhanced receptor binding^35^. Our next step was to determine the structure of the receptor/virus complex. As a control, the structure of the virus/GCDCA complex was determined first. Surprisingly, the bile salts caused a rotation and contraction of the P domains onto the shell domain. This demonstrated that the P is not just a flexible protrusion but that it responds to environmental signals associated with passage in the intestinal lumen. As suggested by the conservation of this feature among Caliciviruses (Figure 2), this P domain flexibility mediates a biological function. In PBS buffer (without metals or bile) at pH 7.4, the shell density was well ordered and easily traced while the P domain was remarkably mobile and highly disordered^22,23,29,30^. When bile was added, the P domain rotated by ~90° and dropped onto the shell (Figure 3). This stabilized the P domain position, and, for the first time, we were able to determine the structure of the entire capsid to ~3Å^36^.

The bound bile salts were easily observed in the EM density (red spheres), and the A’B’/E’F’ loops were in the ‘closed’ conformation found in the x-ray structure of the P domain/CD300lf/GCDCA complex^35^. Bile was not found in any other place in the capsid (e.g., at the P domain/shell interface) and therefore the mechanism of bile-induced P domain contraction was unclear. Nevertheless, these results showed that metabolites in the gut cause the virion to undergo large conformational changes correlated with enhanced receptor binding. In subsequent studies, we also examined the structure of MNV1 at other conditions found in the gut, namely low pH. As shown in Figure 3, the structure of MNV1 at pH 5.0 was identical to when bile was bound. In addition, other researchers found MNV1 was also in this contracted state in the presence of 1 mM calcium^37^. Therefore, three conditions unique to the gut (high bile, low pH, and high metal concentrations) all caused contraction of the P domains onto the shell to yield a conformation optimal for receptor interaction^35^.

## Conformational changes within the P domain

**Figure 4:**
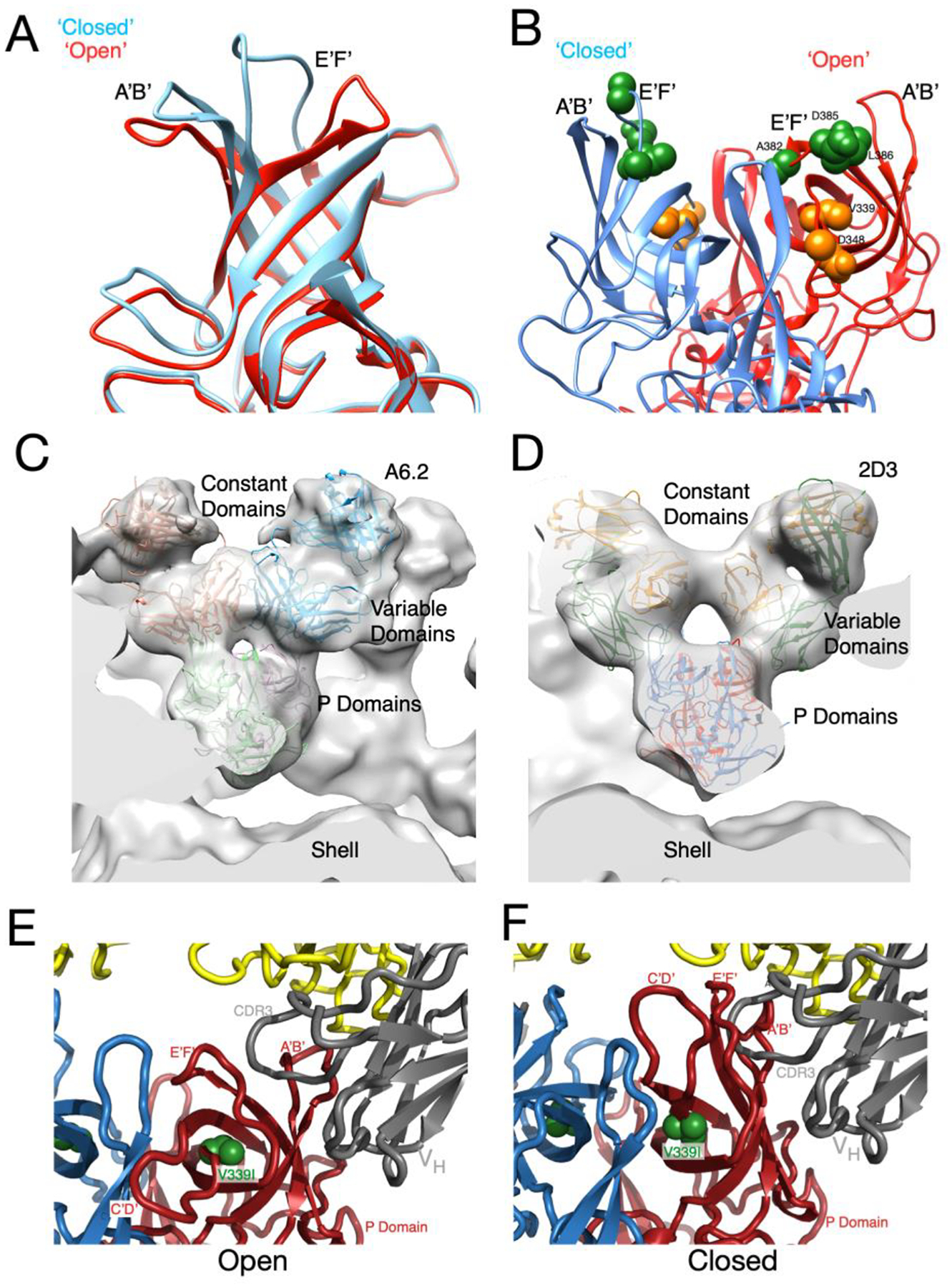
Loop conformational flexibility and antibody recognition. A) The original P domain crystal structure had two different conformations for the A’B’/E’F’ loops; open and closed. B) The escape mutants for A6.2 were found at the very tip of E’F’ (green spheres) while 2D3 was found in the heart of the P domain (orange spheres). C) In spite of the differences in escape mutation location, 2D3 and A6.2 bound to very similar regions. E and F) The pseudo atomic models of the P domain/antibody complexes strongly suggested that antibodies could only bind to the open conformation.

In the original X-ray structure, the loops at the tip of the P domain adopted two different conformations (Figure 4A) where the A’B’ and E’F’ loops were either splayed apart (open) or tightly associated (closed)^30^. In subsequent studies, two different monoclonal antibodies, A6.2 and 2D3, were studied to better understand the mechanism of antibody mediated neutralization^31,32,38^. Interestingly, the escape mutations for these antibodies were non-overlapping and in rather disparate locations (Figure 4B). The escapes for A6.2 (green spheres) are located at the tip of the E’F’ loop while those for 2D3 (orange spheres) are deep within the P2 domain. It was therefore rather surprising that the structures of the whole virus bound with Fab fragments from monoclonal antibodies A6.2 and 2D3 were found to be remarkably similar (Figures 4C, D). Even more unusual was the fact that none of the escape mutations to 2D3 contacted the bound antibody.

Pseudo-atomic models were built of the complexes by fitting the crystal structures of the P domain and the Fabs (Figures 4E, F) into the cryo-EM envelopes to better understand whether the antibodies preferred the open or closed conformations found in the original X-ray structure. As shown in Figure 4E, when the P domain is in the open conformation, there is more than sufficient space to accommodate the CDR3 loop of the bound antibodies. The green spheres show the location of one of the ‘allosteric’ escape mutations to 2D3, showing it is not at all in contact with the antibody. In contrast, the tight association of the A’B’/E’F’ loops in the closed conformation does not afford any space for antibody (Figure 4F). These structures suggested, but could not prove, that the antibodies preferred the ‘open’ conformation. Therefore, we knew there were conformational changes within the P domain and the position/flexibility of the P domain dimer, but lacked information about the structural details, control, or biological relevance.

## High resolution Fab/P domain structure

These results suggested, albeit did not prove, that antibodies prefer the ‘open’ conformation^29,31,32,38^. However, high resolution details were required to prove that antibodies only recognized the ‘open’ conformation. The mobility of the P domain under those conditions precluded obtaining high resolution structures of the antibody complexes in the context of the whole capsid and Fabs were not observed to bind to the MNV/bile complex in cryo-EM reconstructions. Therefore, we expressed the soluble form of the P domain without the shell domain, added Fab fragments from A6.2, and determined the cryo-EM structure of the complex to 3.2Å^36^. As we had hypothesized, the H chain CDR3 loop ‘unfurls’ to reach down into the hydrophobic cleft between the A’B’/E’F’ loops in the ‘open’ conformation. Therefore, this structure demonstrates that the antibodies can only recognize the open conformation at the tip of the P domains.

## Flexibility within the P domain

**Figure 6:**
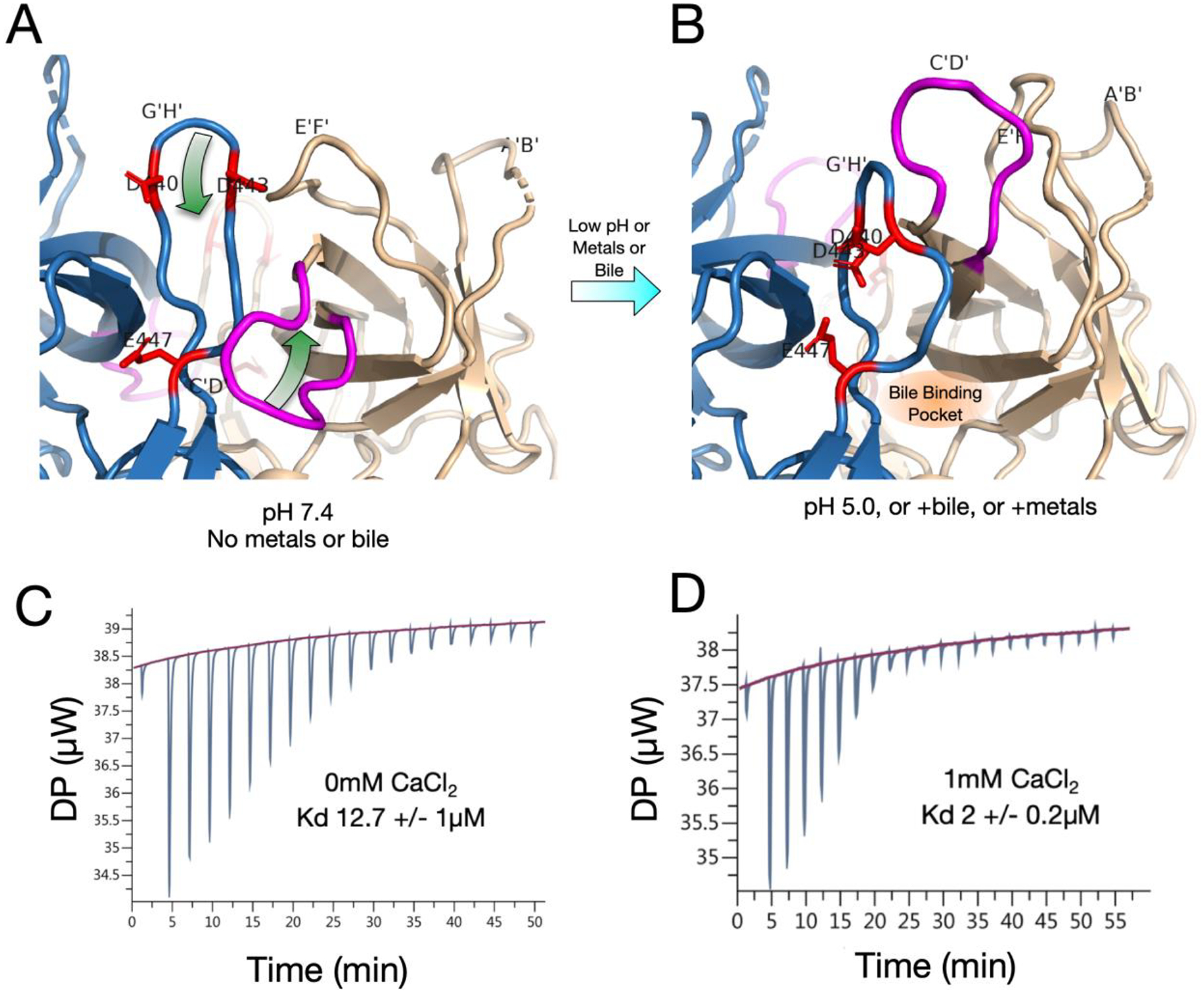
Conformational changes within the P domain during activation. A) Ribbon diagram of the P domain loops at pH 7.4, without any activation triggers. The green arrows indicate the motion of the G’H’ and C’D’ loops upon activation. B) The structures of the P domain loops after being triggered by low pH, metals, or bile salts. C) ITC analysis of GCDCA binding at pH 7.4 in the absence of metals. D) ITC with the same samples and conditions as in (C) except for the addition of 1 mM CaCl_2_.

These results show that gut stimuli (e.g. bile salts, metals, and low pH) cause very large conformational changes in the capsid that result in the contraction of the P domain onto the shell. In these next studies, we examined what conformational changes occur within the P domain as the virus enters this activated state. For this analysis, we compared cryo-EM and crystal structures of the P domain under a wide range of conditions in various complexes^36,39,40^. In the absence of metals and bile at neutral pH, the C’D’ loop points down toward the capsid surface and covers the entrance to the bile binding pocket (Figure 6A). This position opens the area at the tip of the P domain, allowing for the A’B’ and E’F’ loops to splay apart in the open conformation. In the presence of bile, low pH, or metals, the C’D’ loop moves up and pushes the A’B’/E’F’ loops into the closed conformation. While one could envisage bile binding causing C’D’ displacement, it was not immediately clear how low pH or metals could do the same. We proposed this is mediated by the immediately adjacent G’H’ loop^40^. The G’H’ and C’D’ loops essentially swap space at the tip of the P domain switching back and forth from the activated conformation. When one loop is up, the other is necessarily down (Figure 6). At neutral pH, a cluster of acidic groups (i.e. E447, D440, and D443) on the G’H’ loop is expected to be charged and repulsive. This forces the loop into a more vertical orientation and the C’D’ loop folds down to cover the bile binding pocket. However, at acidic pH’s or in the presence of metal ions, the charges are neutralized, allowing the sidechains to interact, and forces the loop down into a compact structure. This, in turn, forces the C’D’ loop up and pushes the A’B’/E’F’ loops into the closed conformation that blocks antibody binding while favoring receptor binding.

If true, one consequence of this model is that access to the bile binding pocket would be enhanced by opening the bile binding pocket by forcing the C’D’ loop into the ‘up’ position. Indeed, this is the case. At neutral pH, the C’D’ loop is expected to be in the ‘down’ position and the Kd for GCDCA using isothermal titration calorimetry (ITC) was determined to be ~13 μM (Figure 6C).

The addition of calcium contracts the G’H’ loop^35^ that in turn lifts the C’D’ loop off the entrance to the bile binding pocket. This increases the binding affinity of GCDCA more than 6-fold to 2 μM (Figure 6D). This lends strong evidence to our model for loop motion and suggests that, *in-vivo*, the metals, bile salts, and metals all act synergistically to prepare the virus for receptor binding.

## Effects of activation triggers on antibody recognition

**Figure 7:**
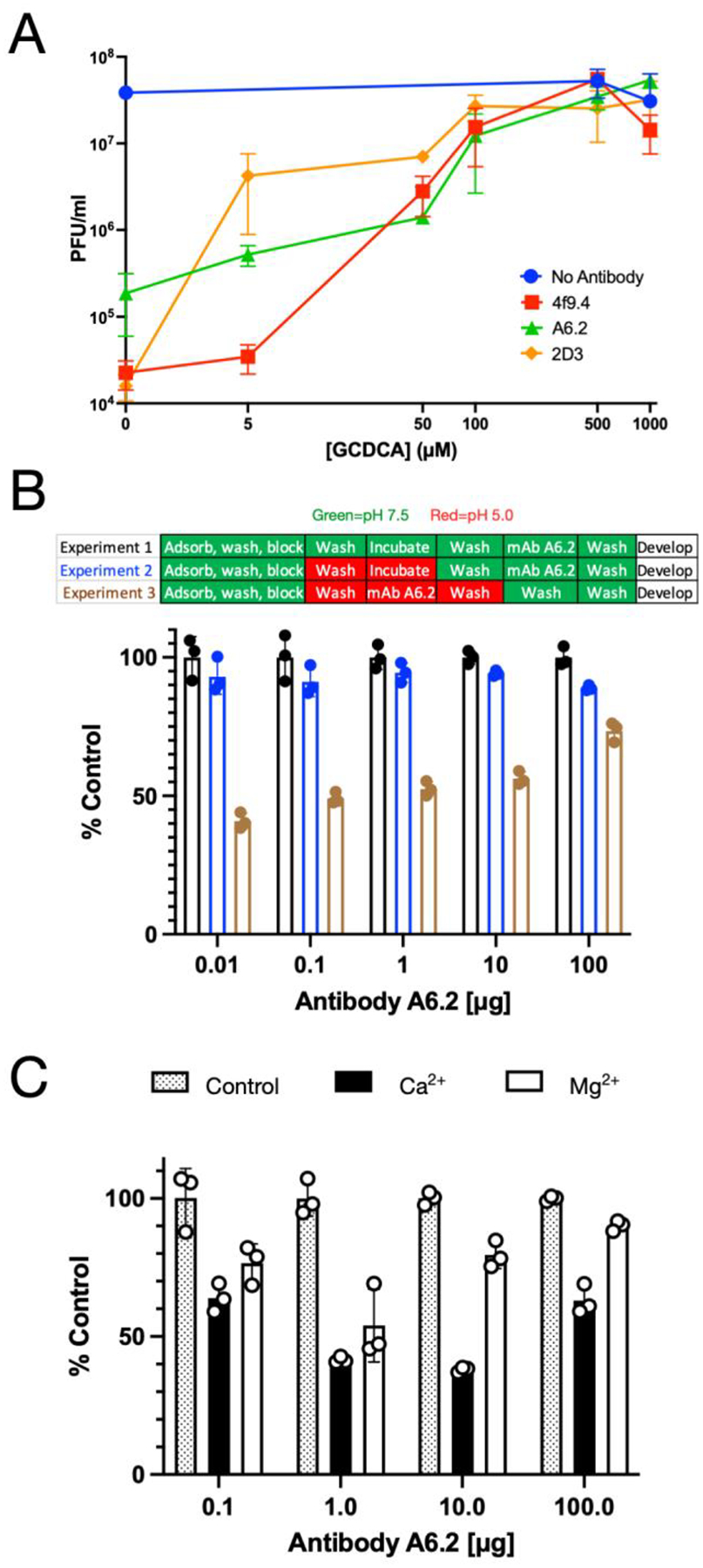
Effects of activation triggers on antibody recognition. A) Plaque assay results showing GCDCA abrogates antibody neutralization of three antibodies in a dose-dependent manner. B) Using ELISA methods, low pH conditions block antibody binding in a reversible manner. The conditions of the three experiments are shown in the bar above the graph. The Y axis represents % of experiment #1 C) ELISA assays showing that metals also block antibody biding. The Y axis represents % of the control of PBS buffer without metals.

Thus far we have shown that the antibodies can only bind to the open conformation and that all the activation signals push the conformation of P domain towards the closed structure. It follows these signals would all block antibody binding. This is indeed the case as shown in Figure 7. Using plaque assays, Figure 7A shows that increasing GCDCA concentrations blocks neutralization by all three monoclonal antibodies in a dose dependent manner. For measuring pH effects (Figure 7B), ELISA assays were used to prevent artifacts in the plaque assay due to cytotoxic effects of acidic conditions on cell monolayers. The black bars show a standard ELISA assay using whole virus as the antigen. There was concern that low pH might disrupt virions and therefore a control experiment was performed (blue bars) to ensure that the pH effects are reversible. Here, the virus was adsorbed to the plates, the plates were blocked with BSA, the virus was treated with low pH buffers for an hour, washed with neutral pH buffers and then the plate was developed as in the first experiment. We know that under these conditions the virus contracts the P domains and the loops adopt the closed conformation at low pH. This control experiment shows that this activation process is completely reversible. Finally (brown bars), if the virus is treated with pH 5.0 buffer on the ELISA plate, antibody is added at those acidic condition, incubated for an hour, washed with low pH buffer, and then developed as above, antibody binding is blocked. As more antibody is added, this diminished affinity is slightly overcome. Similarly, the effects of metals on antibody binding are shown in Figure 7C. Antibody binding is inhibited by the addition of either calcium or magnesium. Therefore, our structural results show that all three environmental signals shift the structure of the P domain towards the closed conformation, and this is concomitant with abrogation of antibody binding. This is opposite to the receptor interactions that are enhanced by metals and bile salts^35^. This shows that, once in the gut, the virus changes its structure from one that is recognized by antibodies to one that is optimized for receptor binding.

## Structural mechanism of P domain contraction

**Figure 8:**
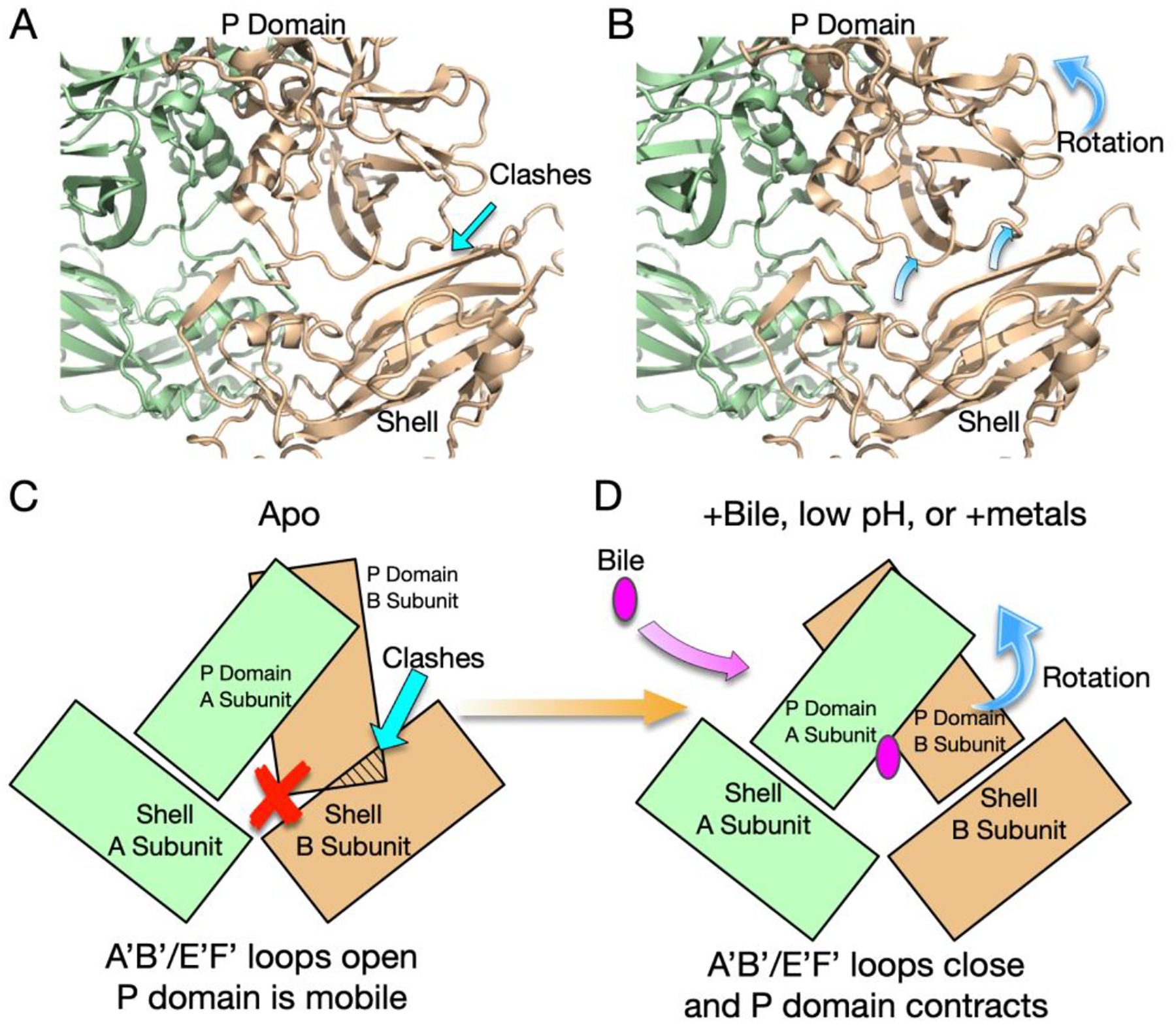
P domain rotation as a possible switch for contraction. A) The apo structure of the P domain alone aligned onto the contracted whole virus structure. As noted by the cyan arrows, this causes extensive clashes between the base of the P domain and the shell and is the reason why the P domain ‘floats’ above the shell in the absence of activation triggers. C,D) Schematic of this motion showing that the apo structure cannot contract onto the shell surface but forms a complementary surface upon activation.

One of the major mysteries was how these metabolites binding at the top of the P domain could cause contraction of the P domains onto the surface of the shell. When comparing the various EM and X-ray structures of the P domain, there appeared to be a large rotation of the subunits when comparing the extended (‘floating’) P domain with the open loop structure to the contracted P domain with the closed loop conformations^36,40^. Since the core of the P1 domain itself (residues 500–530) is unaffected by the conformational changes in the loops in the P2 domain, structural alignments were performed using only this region. If the relationship between the A and B subunits are unaffected by the activators (i.e. low pH, metals, or bile), then the P1 domains of the B subunits would be expected to align as well as the fitted P1 domains of the A subunits. However, what is evident from this alignment is that bile, low pH, or metal binding causes the A/B dimers to rotate with respect to each other (Figure 8). Most importantly, when the aligned P domain structure without bile was placed onto the shell using this P1 domain alignment, extensive clashes with the shell are apparent. Indeed, without bile, there are five times more contacts between the B subunit and the shell that are less than 3.5Å and 21 contacts are less than 2Å. As shown by the arrows in Figure 8, bile causes the subunits rotate and create an optimal surface for contact with the shell domain. This motion is summarized in the cartoon in Figure 8C, D. Once the P domains have rotated about each other, the base of the P domain dimer becomes complementary to the shell surface and the P domains contract onto the shell. It is not wholly surprising that these activation conditions, even though they are affecting the loops at the tip of the P domain, all cause rotation of the P domains about each other. The loop movement shown in Figure 6 is right at the dimer interface and the loops in motion are intertwined. In this way, all three rather disparate activation signals can all cause the same contraction and loop structure.

## Ballet of motion in MNV during activation

**Figure 9:**
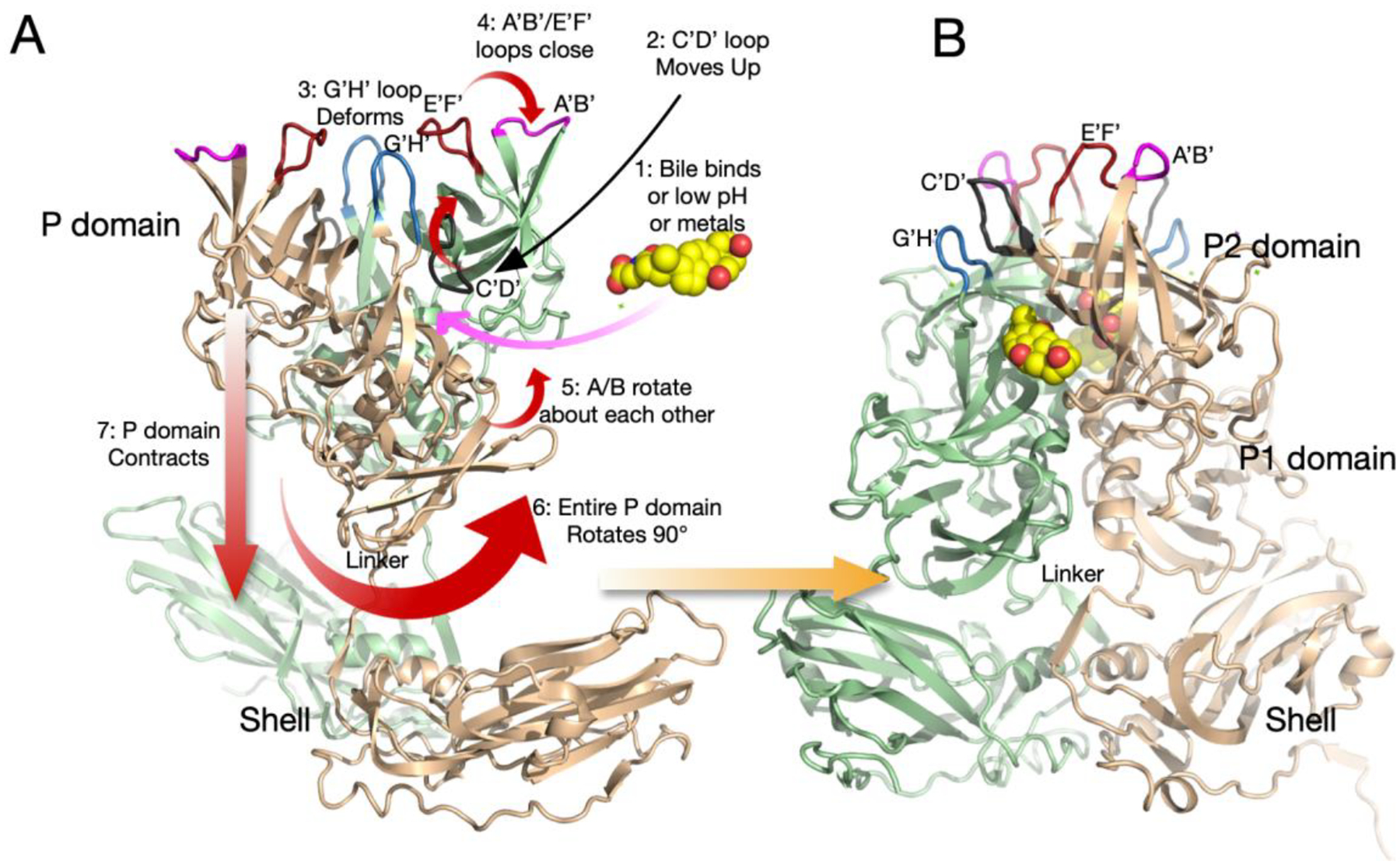
Summary of the conformational changes necessary to activate the MNV capsid. A) The apo structure of MNV. The arrows show all the changes necessary to close the A’B’/E’F’ loops and to contract the P domain onto the shell surface.

Figure 9 reviews the steps in the structural transition from the apo (floating P domains, open A’B’/E’F’ loops) to the contracted/closed virion structure. At pH 7.5, in the absence of bile and metals, the C’D’ loop is in the down position and the A’B’/E’F’ loops are splayed apart in the open conformation. Since the C’D’ loop has not moved upwards under these conditions, the A/B subunits have not rotated and therefore the P domain cannot associate with the shell. The transformation to the contracted state starts with the addition of bile, low pH, or metals (Figure 9, #1). Bile may directly move the C’D’ loop upwards (Figure 9, #2) by binding directly beneath it. Alternatively, metals and low pH conditions may move the C’D’ loop indirectly by distorting the G’H’ loop and filling the space normally occupied by the C’D’ loop (Figure 9, #3). We propose that the movement of the C’D’ loop then causes the A’B’/E’F’ loops to close (Figure 9, #4) and to rotate the A/B subunits about each other (Figure 9, #5). Once the A/B subunits have rotated, the surface at the base of the P domain changes to form a complementary surface to the top of the shell. Now that the P domain can bind to the shell, the flexible linker allows the P domain to rotate by ~90° (Figure 9, #6) and contract onto the shell (Figure 9, #7). While this figure shows a stepwise process, clearly these movements all must occur cooperatively. It is important to note that this summary is constructed from several structures using both crystallographic and cryo-EM methods that all lead to the same conclusions. What is particularly interesting is that one of the two escape mutants to the neutralizing antibody, 2D3, is the buried residue V339 that lies immediately adjacent to the bile salt binding site. As with the bile salts, we proposed that the V339I mutation shifted the equilibrium towards the closed conformation that does not favor antibody binding^38,41,42^. Therefore, while mutations deep in the protein core might be expected to have a negative impact on virion viability, this allosteric escape mutant may exhibit improved receptor binding by mimicking the bile, low pH, and metal binding effects while blocking antibody binding.

## Structural mechanism for antibody/receptor switching

**Figure 10:**
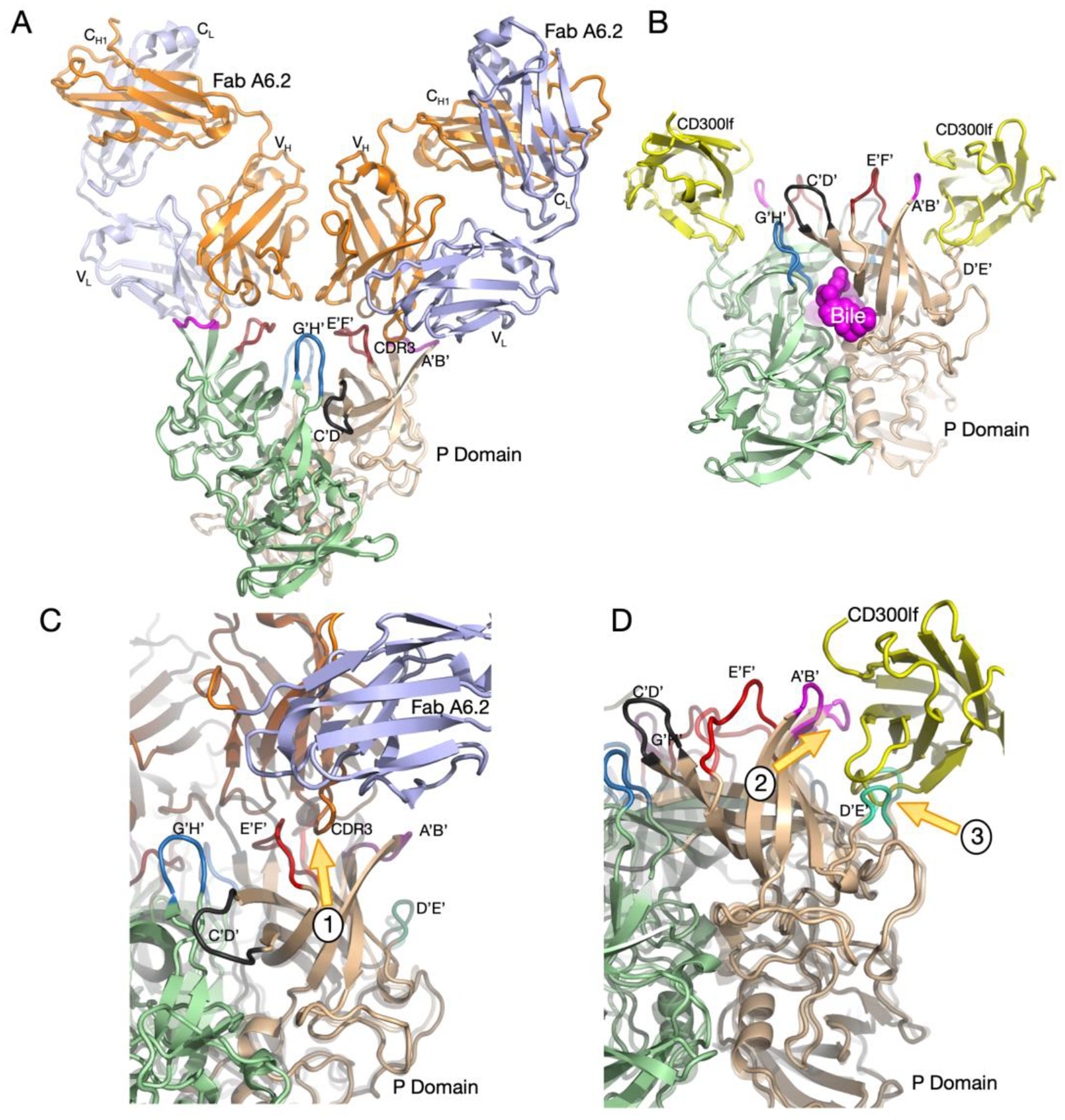
Antibody and receptor binding are mutually exclusive. A and B) P domain complexed with antibody and receptor, respectively. C) The structure of A6.2 bound to the open conformation. The closed conformation is the transparent overlay. D) The structure of CD300lf bound to the closed conformation. The open conformation is shown as a transparent overlay. Clashes are noted by the arrows.

It is sterically impossible for both antibody and receptor to simultaneously bind to the top of the P domain because of their sheer bulk. Additionally, the structure of the P domain itself precludes this as well. Figure 10 shows the structures of the isolated P domains with antibody (Figure 10A) and receptor (Figure 10B) bound. As discussed above, the apo form (floating P domains with open A’B’/E’F’ loops) is the structure recognized by the antibodies. Once the virus is activated by low pH, bile salts, or metals, the A’B’/E’F’ loops close and the antibody is no longer able to bind. The structural basis for this binding abrogation is again illustrated in panel C. Overlaid on the antibody complex in the open conformation is a transparent figure of the closed structure. The orange arrow 1 notes how the CDR3 clashes with the A’B’/E’F’ loops in the closed conformation and thus the antibody is unable to bind to the activated state. In contrast, the CD300lf receptor clearly prefers the closed conformation (panels B and D), consistent with enhanced binding in the presence of activation triggers. In the apo, open state (transparent image), the A’B’ loop is splayed open, away from the E’F’ loop. In this position, the sidechains in the A’B’ loop are far too close to the bound CD300lf receptor (Figure 10D, arrow 2). Similarly, the D’E’ loop of the P domain in the open conformation overlaps the bound receptor but moves away upon activation (arrow 3). Therefore, the reversible switching between antibody and receptor binding are entirely controlled by the position of all these loops at the tip of the P domain that are in turn controlled by the presence/absence of activation triggers (i.e. bile salts, metals, and low pH).

## *In-vivo* structural/functional changes are tissue dependent.

**Figure 11:**
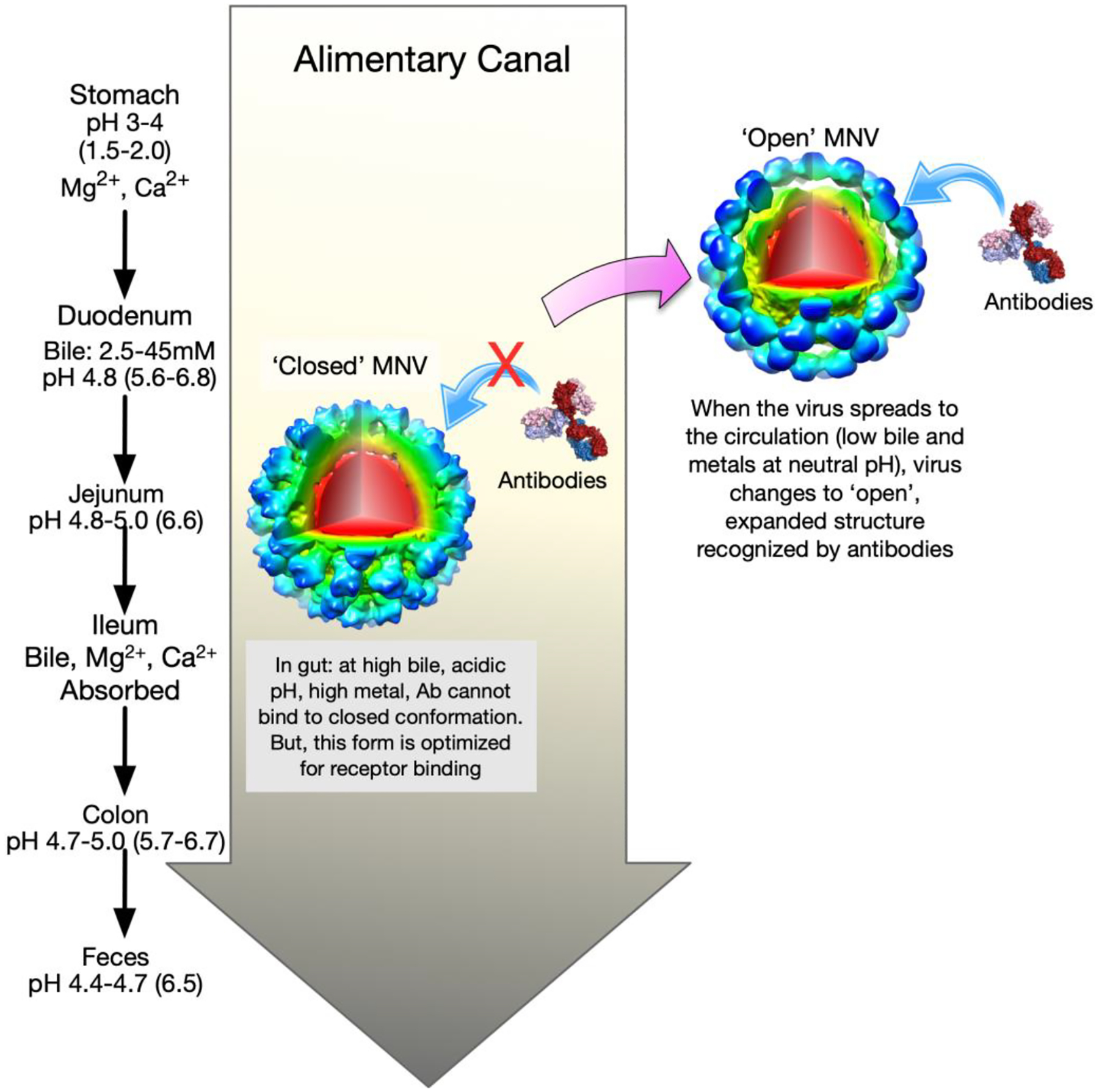
Shapeshifting MNV presents different conformations in the gut and in the circulatory system. Shown on the left are the approximate conditions found in the alimentary canal in mice with human conditions noted in parentheses. Throughout the intestines, the metal, bile, and pH triggers are expected to keep it in the activated state that does not bind antibodies. Once the infection spreads to the circulation, MNV adopts the ‘floating P domain’ structure with open A’B’/E’F’ loops to which antibodies bind.

Putting all these results into the *in-vivo* context demonstrates how stealthily MNV avoids the immune system. It is apparent that when the virus enters the gut it reversibly changes its ‘face’ from one recognized by antibodies to one with enhanced receptor binding properties (Figure 11). As the virus enters the stomach, it is immediately exposed to highly acidic conditions that will cause the P domains to contract and the A’B’/E’F’ loops to adopt the closed conformation. In addition, the low pH increases the solubility of metals like calcium and magnesium (e.g.^43^) that will also cause contraction. While we have shown above that this conformation blocks antibody binding while enhancing receptor binding, it is entirely possible that it serves other purposes as well. Perhaps this contracted structure is more resistant to proteases and pH denaturation. It could be akin to a turtle, tucking in the P domain to protect it from the harsh conditions in the stomach. Once in the duodenum, the pH increases slightly but is now exposed to high concentrations of bile. The three activation signals (low pH, metals, and bile) are maintained throughout the small intestine until the metals and bile are absorbed in the ileum. However, the low pH is maintained throughout the intestine all the way to the feces. Again, perhaps this contracted state is more stable and thus best suited for spread to the next host and that the virus is in the expanded form as it moves through the tissue or in cell culture. From our structure of the whole virus/receptor complex, we also suggested that perhaps there is room for more copies of CD300lf to bind while the whole virus is in the contracted form^35^. Therefore, the triggers may improve avidity for the receptor^23,35^. Therefore, throughout its passage in the gut, this contracted/closed form of the virus is optimized for infecting its target tissue while letting the host’s metabolites block antibody recognition. Once departing the gut environment and entering the circulation, all three activation signals are removed, and the virus converts to the ‘floating P domain’ structure with open A’B’/E’F’ loops. Therefore, what is presented to the immune system is unrelated to the activated particle in the gut. It should be noted that a fraction of the results reviewed here has been recapitulated in GII.4 human norovirus in a very recent publication^44^. For reasons that are not clear, the authors appear to be unaware of the highly relevant results and conditions presented here that had been published over the past decade.

To our knowledge, this is the first example of a virus using host metabolites to reversibly change its antigenic face to thwart the immune response. From our structures, the immune system recognizes the ‘floppy’ P domain with the epitope in the ‘open’ conformation. Once it enters the gut, the epitope closes, and the P domain contracts onto the shell. This structure is no longer recognized by antibodies made in the circulation but is optimized for cell binding. Recently, at least two groups have shown similar immune evasion during SARS-CoV-2 infections. In the first publication, the authors found that heme breakdown products bind to the spikes and block antibody binding^45^. In a more recent publication, authors suggest that free fatty acids found at sites of inflammation might similarly diminish antibody neutralization efficacy^46^. Therefore, our finding is highly relevant to other disparate viral systems and has importance in vaccine development.

## Conclusions:

Discussions of viral immune avoidance mostly focus on locations and effects of escape mutations that block antibody binding. For most viruses, this is the major concern as we adapt and improve our vaccines. However, as reviewed here, we are finding more instances whereby the viruses are actively evading antibody recognition by hijacking host metabolites. It is becoming more apparent that viruses are indeed flexible, dynamic structures and thusly can morph into new conformations with relatively low energy inputs from their environment. Therefore, in many cases, we will need to consider not just genetic plasticity but conformational as well.

## Figures and Tables

**Figure 5:**
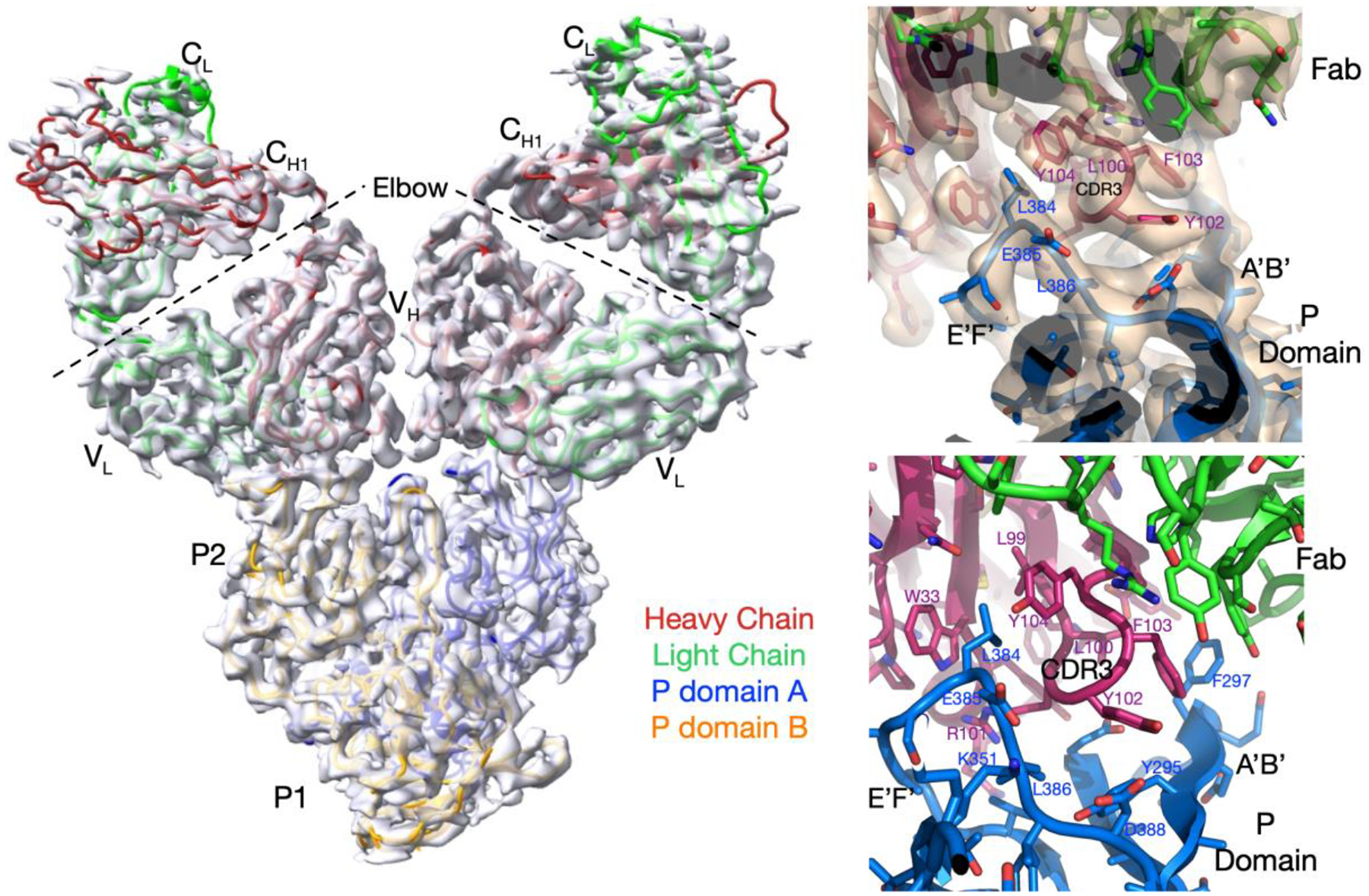
High resolution structure of the A6.2/P domain complex. At left is the cryo-EM envelope and refined structure of the entire Pdomain/A6.2 complex. At right is a closeup of the paratope-epitope contact. The A’B’/E’F’ loops are clearly in the open conformation and the CDR3 loop reaches deeply into the cleft for binding.
